# Supplementary material for: Exploring the density and morphology of coconut structures at two locations: a time-based analysis using computer tomography
Source: PeerJ. 2024 Oct 14;12:e18206. doi: 10.7717/peerj.18206 (PMC11485131; doi:10.7717/peerj.18206)
Supplement: Supplemental Information 1 — The CT values of various parts of the coconut, including the embryo, bud, solid endosperm, coconut water, mesocarp, endocarp, and coconut apple. [file peerj-12-18206-s001.pdf]

The following are the test materials, test images, and data record tables for coconut CT testing. Specifically, as follows:

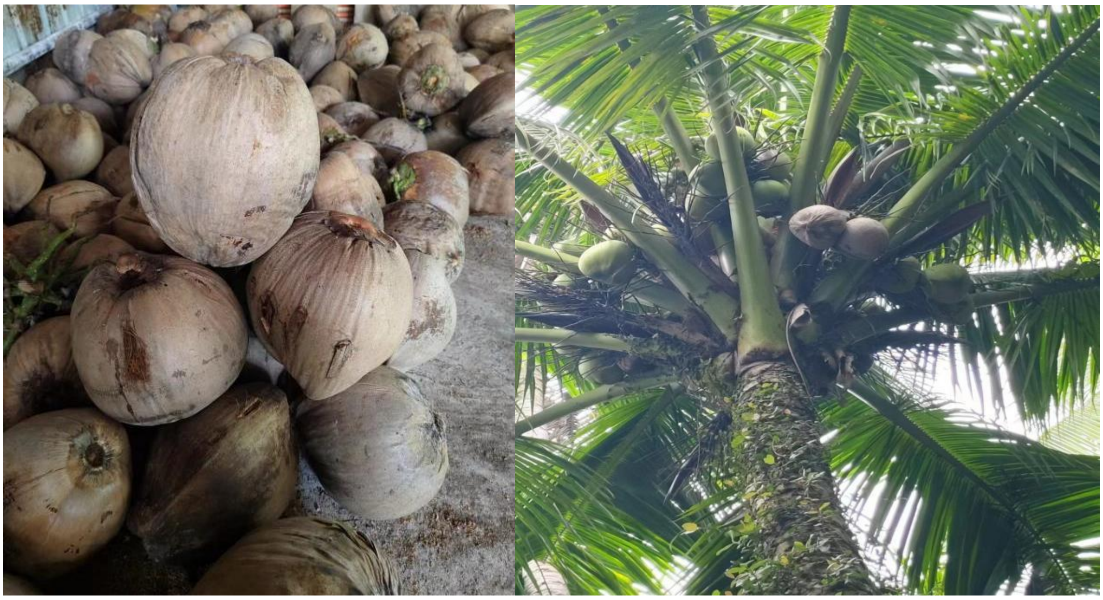

Figure S1: Coconut material tested

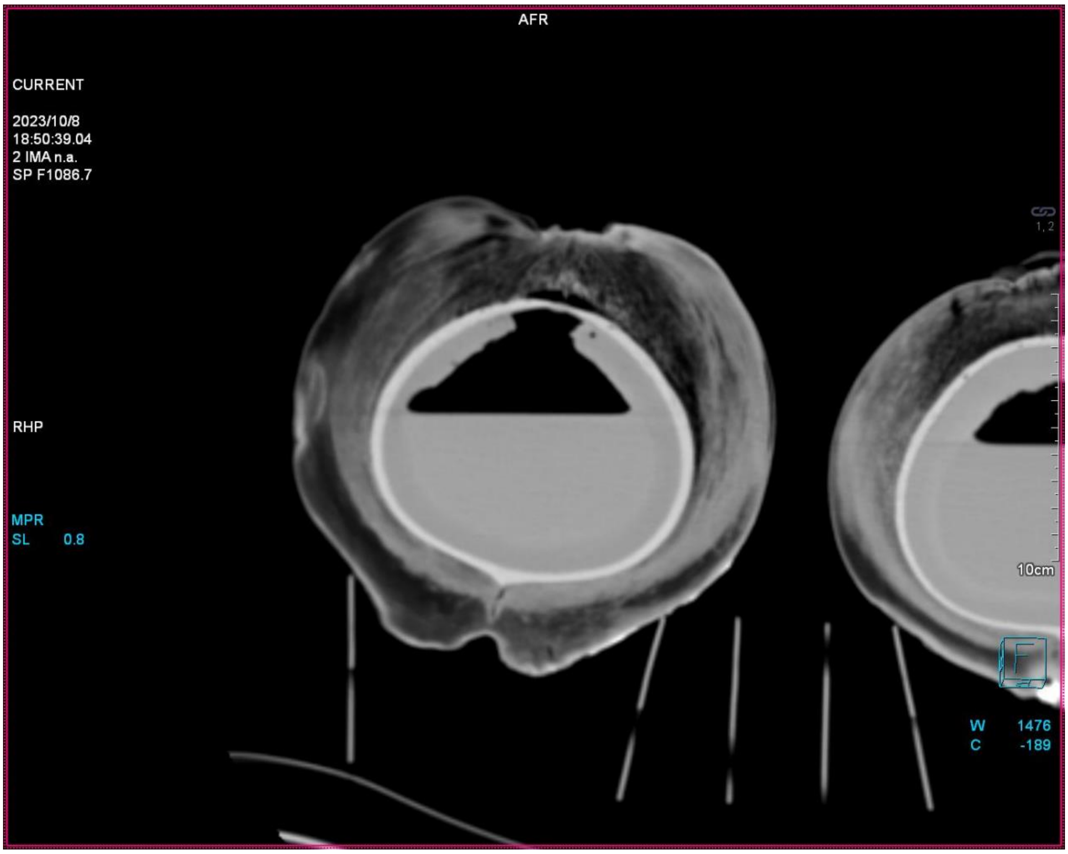

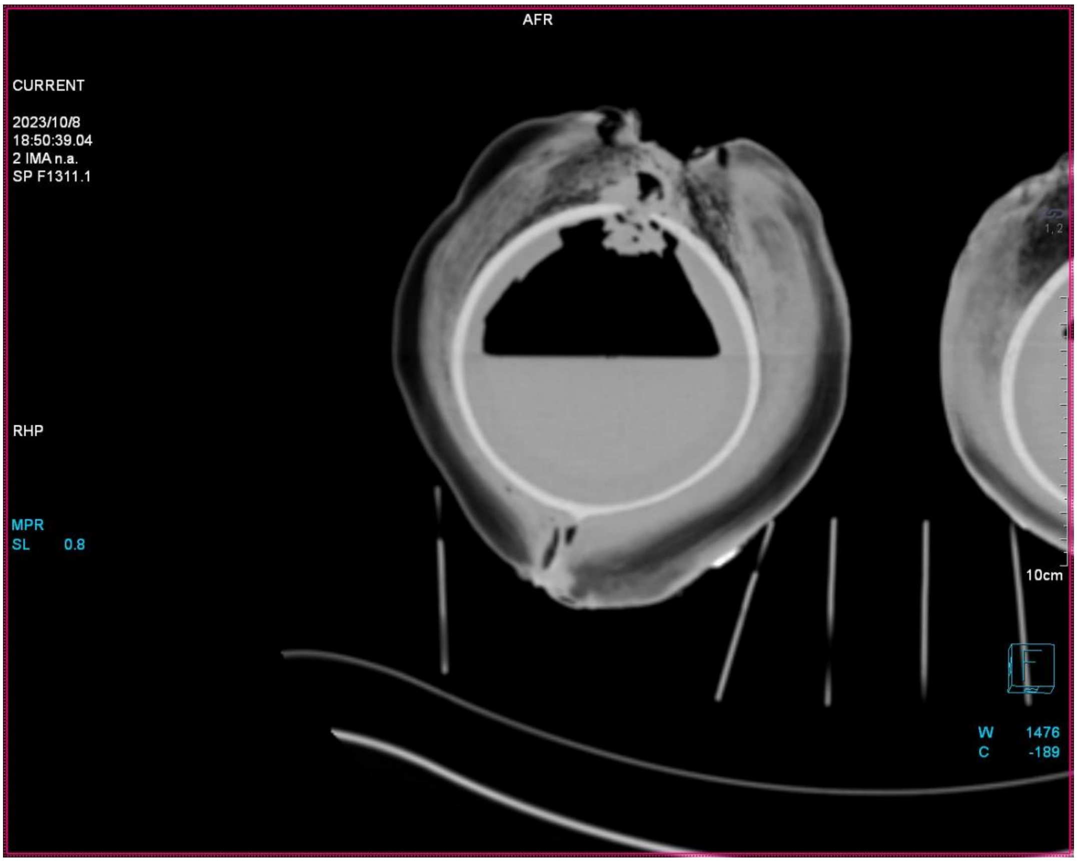
AFR
CURRENT
2023/10/8
18:50:39.04
2 IMA n.a.
SP F1311.1
RHP
MPR
SL 0.8
10cm
W 1476
C -189

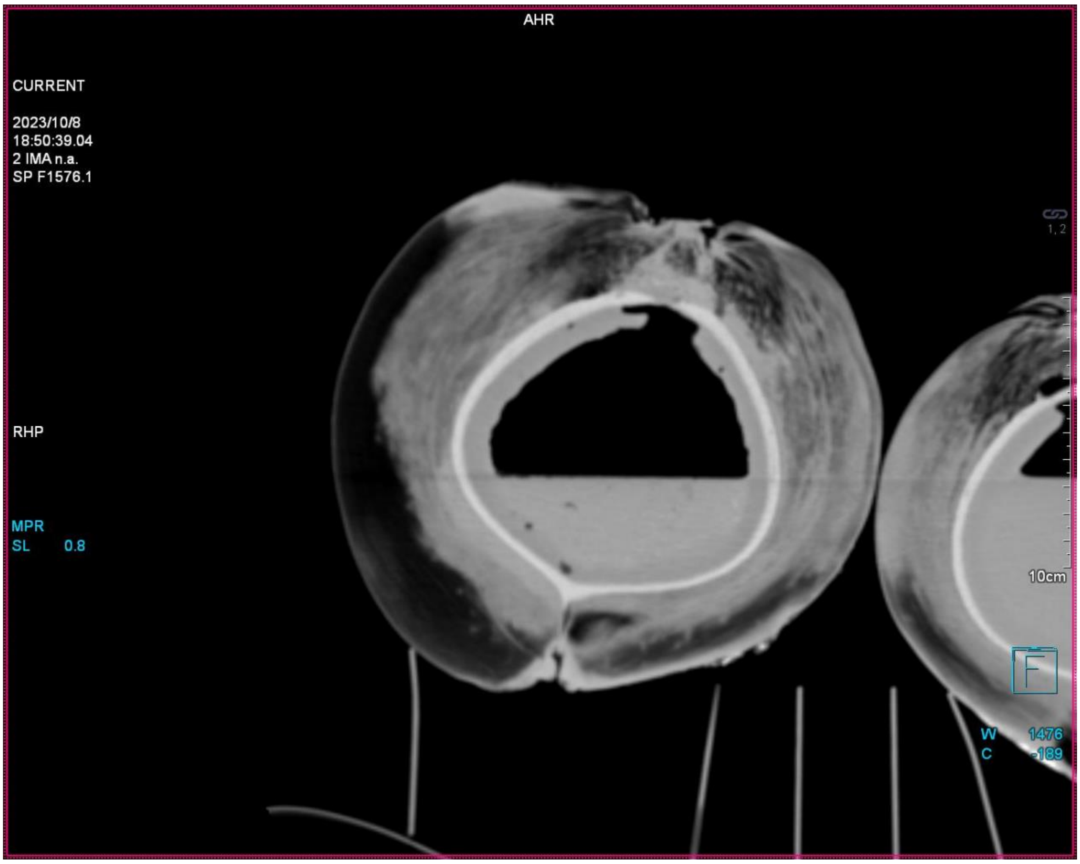
AHR
CURRENT
2023/10/8
18:50:39.04
2 IMA n.a.
SP F1576.1
RHP
MPR
SL 0.8
10cm
W 1476
C -189

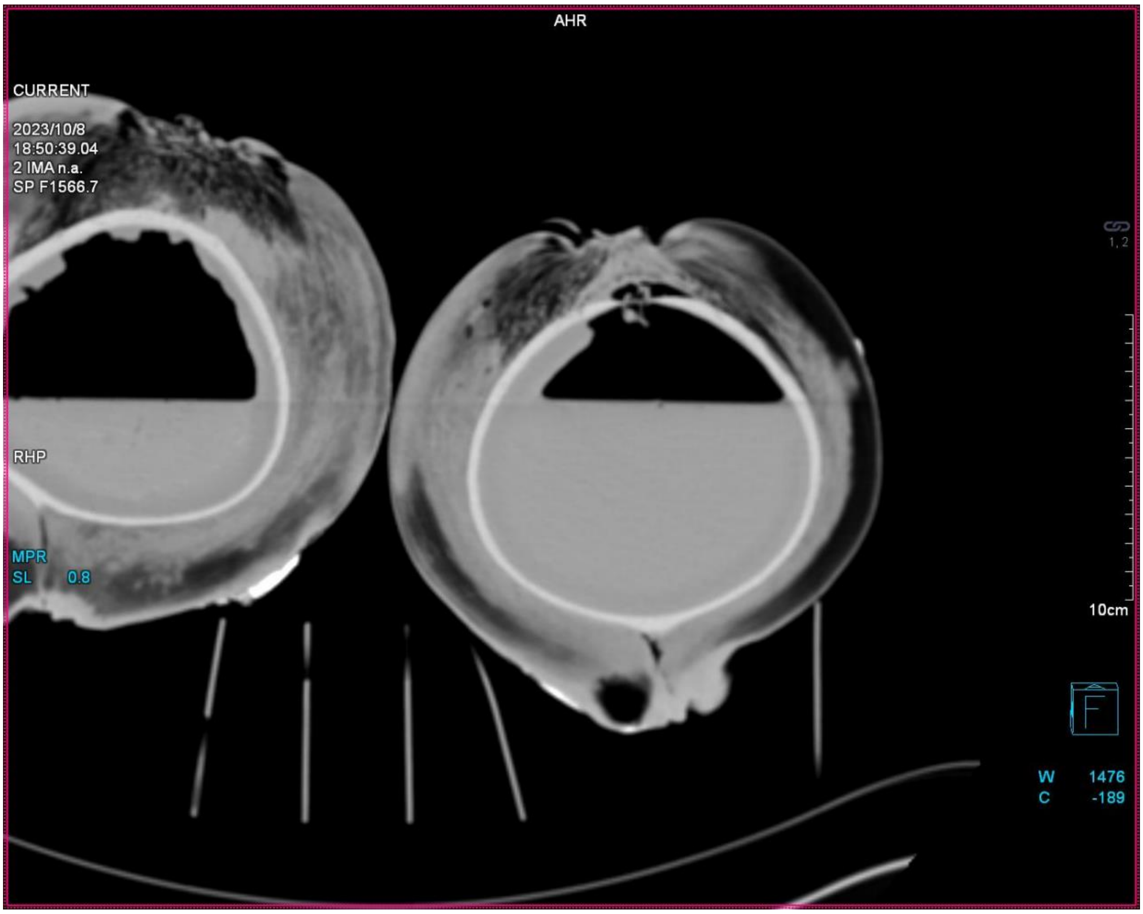

Figure S2 CT images of some coconut fruits

Table S1 CT test coconut data record table

CT value record table for each organization															
ldentifier	Eembryo	Bud	Coconut meat			Coconut shell (inner skin)			Coconut fiber (mesocarp)			Coconut water			
			1	2	3	1	2	3	1	2	3	1	2	3	
1101-2-4						29	28	33	-15	-11	-8	26	27	23	
1101-2-5						32	33	38	-30	-9	-6	25	24	21	
1101-4-7						27	26	32	-10	9	-16	13	16	25	
1101-4-8						20	30	37	-37	-15	-21	14	24	16	
1101-4-9						26	29	37	-40	-30	-31	20	21	24	
1101-6-4			39	40	55	115	107	105	-87	-85	-53	11	19	17	
1101-6-5			42	55	56	165	139	165	-59	-63	-77	28	23	32	
1101-8-4			43	52	33	103	123	115	-77	-43	-21	28	33	32	
1101-8-5			47	57	43	116	136	142	-101	-98	-67	23	17	29	
1101-10-1			36	38	41	248	186	202	-828	-814	-775	29	26	26	
1101-10-4			30	32	33	220	136	219	-827	-803	-725	19	25	28	
1101-12-4			18	45	22	172	155	174	-812	-824	-778	16	14	19	
1101-12-5			24	16	53	149	127	123	-785	-714	-809	19	20	9	
1101-12-6			26	57	61	125	177	76	-742	-726	-697	15	29	28	
